# Effects of Sr/F-bioactive glass nanoparticles on pH, elemental release, dentin remineralisation, and cytotoxicity of 1.1% NaF toothpaste

**DOI:** 10.2340/biid.v12.44239

**Published:** 2025-07-22

**Authors:** Chananya Gesprasert, Matana Kettratad, Parichart Naruphontjirakul, Piyaphong Panpisut

**Affiliations:** a Dental Department, Prasat Hospital, Surin, Thailand; b Faculty of Dentistry, Thammasat University, Pathum Thani, Thailand; c Biological Engineering Program, Faculty of Engineering, King Mongkut’s University of Technology Thonburi, Bangkok, Thailand; d Thammasat University Research Unit in Dental and Bone Substitute Biomaterials, Thammasat University, Pathum Thani, Thailand

**Keywords:** Bioactive glass, 5000 ppm toothpaste, fluoride release, elemental release, cytotoxicity, remineralisation

## Abstract

**Objective:**

This study examined the effect of Sr/F-bioactive glass nanoparticles (Sr/F-BAG) concentration on 1.1% NaF toothpaste. The effects of additives on pH, fluoride and elemental release, dentin remineralisation, and cytotoxicity were determined.

**Materials and methods:**

Sr/F-BAG particles were incorporated into 1.1% NaF toothpaste (0, 1, 2, and 4 wt%). F release and pH upon immersion in deionised water were determined using a fluoride-specific electrode and pH meter (*n* = 8). Elemental release was analysed using Inductively Coupled Plasma Optical Emission Spectroscopy (*n* = 3). Dentin remineralisation (mineral-to-collagen ratio) after application of experimental toothpaste was compared using Attenuated Total Reflectance Fourier Transform Infrared Spectroscopy (ATR-FTIR, *n* = 9). Cytotoxicity was assessed using the 3-(4,5-dimethylthiazol-2-yl)-2,5-diphenyltetrazolium bromide (MTT) assay (*n* = 3). Colgate PreviDent 5000 Plus toothpaste (PV) was used as a commercial comparison.

**Results:**

The addition of 0 to 4 wt% Sr/F-BAG linearly increased pH and F release of the 1.1% NaF toothpaste. Each 1 wt% increase in Sr/F-BAG concentration, raised pH by 0.3 and fluoride release by 457 ppm. The additives also enhanced the release of Ca, P, and Sr from the experimental toothpaste. At high concentration of Sr/F-BAG (4 wt%), the pH of the experimental toothpaste was comparable to PV (*p* > 0.05) but with significantly higher fluoride release (*p* < 0.05). However, PV demonstrated a significantly higher increase in mineral-to-collagen ratio compared to the experimental materials. The dentin surface treated with PV also showed more evident mineral precipitation. Furthermore, the experimental toothpaste containing 4 wt% Sr/F-BAG demonstrated higher cell viability (90%) than PV (56%).

**Conclusion:**

The addition of Sr/F-BAG enhanced the release of F, Ca, P, Sr, and increased the pH of the toothpaste. However, the experimental toothpaste with added bioactive glass up to 4 wt% did not demonstrate superior remineralising effects compared to commercial 1.1% NaF toothpaste. In addition, the incorporation of Sr/F-BAG promoted the cytocompatibility of the experimental toothpaste.

**KEY MESSAGES**

1. The addition of bioactive glass promoted the release of fluoride, calcium, and phosphorus, and also enhanced the pH level and in vitro cytocompatibility of the experimental 1.1% NaF toothpaste.
2. The addition of 1 to 4 wt% Sr/F-bioactive glass nanoparticles to 1.1% NaF toothpaste showed no significant effect on remineralising actions on demineralised dentin.
3. The commercial 1.1 wt% NaF toothpaste (Prevident) exhibited the highest remineralising action compared to other experimental materials.

## Introduction

Dental caries is the most common preventable disease affecting people globally. It was estimated that the disease affected approximately 3.5 billion adults worldwide, which has been increasing in the past few decades [1]. Caries significantly reduces the quality of life, as reported by the 2017 Global Burden of Disease (GBD) study, which estimated that 20.7 age-standardised healthy years per 100,000 people are lost due to caries in permanent teeth [2]. The use of high-concentration fluoride (1.1% NaF toothpaste, 5,000 ppm) is currently recommended for controlling active dental caries in permanent teeth for high caries risk patients [3]. This preventive approach was also in agreement with the World Health Organization (WHO) Bangkok Declaration in 2024, which emphasised that primary dental care should be prioritised in all countries [4].

Fluoride toothpaste is listed as an essential medicine by the WHO [5]. However, a study reported significant inequity in the affordability of fluoride toothpaste among World Bank income groups [6]. The study revealed that the percentage of countries where fluoride toothpaste was unaffordable in high-income and upper-middle-income countries was 0% and 16%, respectively [6]. In contrast, the unaffordable percentages were as high as 72 and 86% in lower-middle-income and low-income countries. Currently, 1.1 wt% NaF toothpaste (5,000 ppm) is regulated as a medical device and requires a prescription from a clinician [7]. The availability of this toothpaste in each country is not well-documented, and sales and production data from manufacturers are not generally publicly available [6]. The lack of product availability and affordability could potentially limit oral health promotion programmes, hindering efforts to reduce the caries burden. The locally produced and simplified formulations of 1.1 wt% NaF toothpaste with desirable remineralising effects could be beneficial for production or use in regions with limited access to commercially available 1.1 wt% NaF toothpaste.

The addition of bioactive glass in toothpaste is expected to enhance remineralisation for controlling caries in high caries risk patients [8]. In various dental applications, bioactive glass has enhanced mineral precipitation on dentin surfaces by promoting hydroxyapatite formation, improving the nano-mechanical properties of demineralised dentin, and providing antibacterial actions [9, 10]. An additional benefit of bioactive glass is its ability to occlude dentinal tubules, which is expected to reduce dentin permeability [11]. This occurs because of the release of calcium and phosphate from the bioactive glass, which then precipitate as calcium phosphate inside the tubules [12]. A previous study prepared experimental 1.1% NaF toothpaste with 1 wt% bioactive glass to enhance remineralisation of irradiated dentin [13]. However, this formulation demonstrated no significant remineralising benefit compared to commercial products [13]. The current study increased the concentration to 4 wt%. Since bioactive glass rapidly exchanges ions with the surrounding environment, leading to increased pH [14], the pH changes and enhanced elemental release may induce bioactive effects [15], which may require toxicity assessment of the toothpaste. It was expected that the fluoride toothpaste with enhanced remineralisation and antibacterial properties would be suitable for patients at extremely high risk of caries, such as severe xerostomia patients. Accordingly, incorporating bioactive glass nanoparticles, which could potentially facilitate the release of ions such as Ca, Sr, and F, offers dual benefits in application and contributes to the prevention of dental caries.

The aim of this study was to determine the effect of concentration of bioactive glass nanoparticles (1–4 wt%) on the pH, fluoride release, elemental release, remineralising actions, and cytocompatibility of the experimental 1.1% NaF toothpaste. The null hypothesis of the study was that the addition of bioactive glass nanoparticles should not exhibit a significant effect on pH, fluoride release, elemental release, remineralising actions, and cytocompatibility of the experimental toothpaste. In addition, the tested properties of the experimental materials should not significantly differ from those of the commonly used commercial 1.1% NaF toothpaste.

## Materials and methods

### Materials preparation

Sr/F-doped bioactive glass nanoparticles (Sr/F-BAG) were synthesised using a modified sol-gel method incorporating post-functionalisation and heat treatment processes, following previously established protocols [16]. Briefly, silica nanoparticles (SiO_2_–NPs) were prepared prior to incorporating with Ca, Sr, and F to obtain Sr/F bioactive glass nanoparticles. Firstly, 61.7 mL of deionised water, 7.2 mL of ammonium hydroxide (Merck, Darmstadt, Germany), and 493.8 mL of ethanol (Merck, Darmstadt, Germany) were mixed in a 1 L Erlenmeyer flask at 600 rpm for 30 min using a magnetic stirrer. Thereafter, 37.5 mL of tetra orthosilicate (TEOS, Sigma-Aldrich, St. Louis, MO, USA) was gently added and stirred overnight at room temperature to complete hydrolysis and condensation reactions, resulting in silica network formation. SiO_2_-NPs were collected using a centrifuge at 5,000 rpm for 30 min and washed with deionised water. The obtained SiO_2_-NPs were resuspended in deionised water. Then, 12.9 g of Ca(NO_3_)_2_·4H_2_O, 34.7 g of Sr(NO_3_)_2_, and 0.9 g of NaF (Merck, Darmstadt, Germany) were added with a nominal molar ratio of SiO_2_: CaO:SrO: NaF at 1.0:0.33:1.0:0.5.

**Figure 1:**
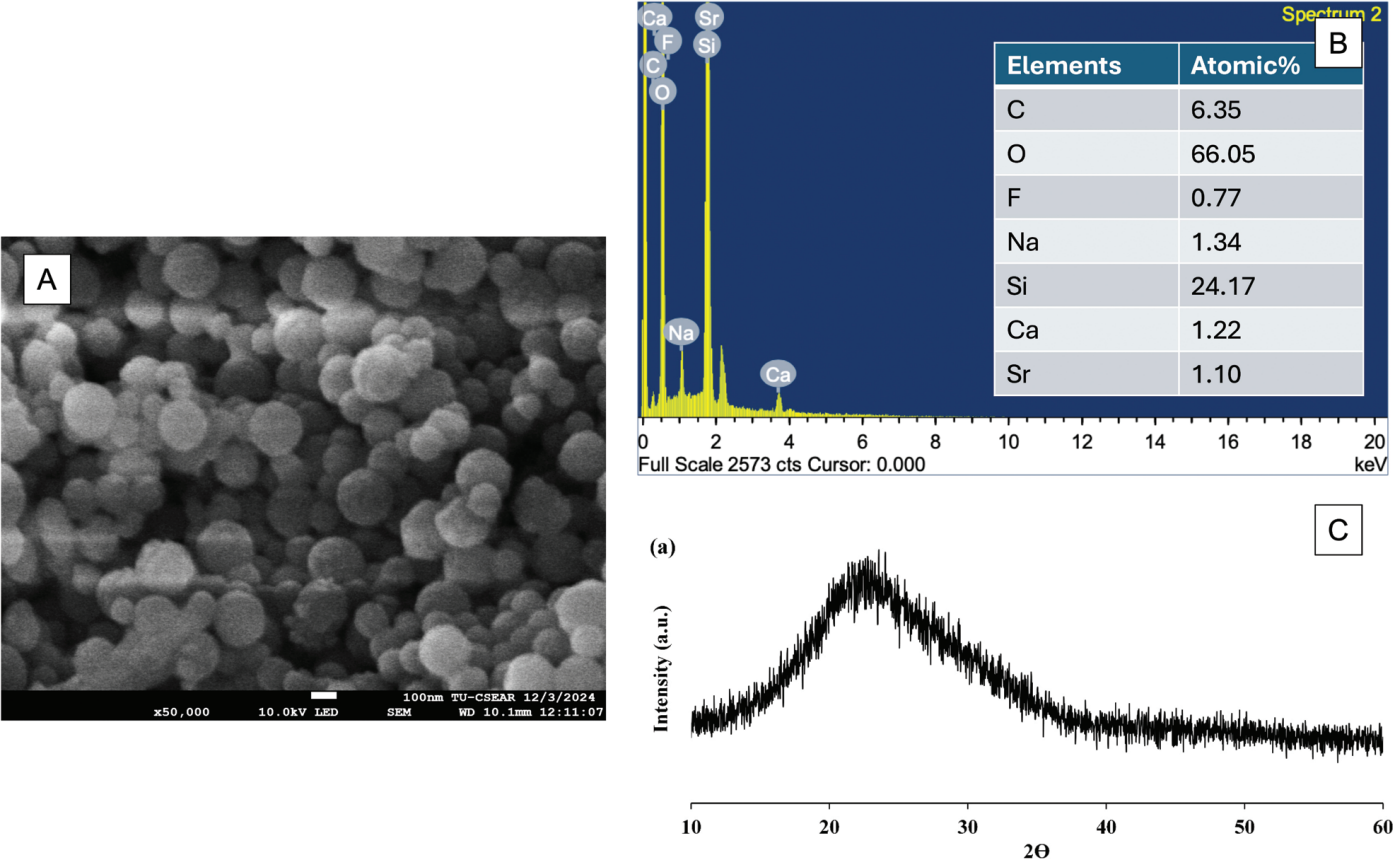
(A) The SEM image of Sr/F-BAG and (B) The result from the EDX analysis. (C) The result from the XRD pattern of the glass. Figure 1-C was reprinted from the reference [16] under the Creative Commons Attribution (CC BY) license.

The diameter of the obtained glass particles was approximately ~200 nm [13, 17]. The presence of remineralisation-enhancing elements, including Ca, Sr, and F, was confirmed using scanning electron microscopy (SEM, JSM 7800 F, JEOL, Tokyo, Japan) coupled with energy-dispersive X-ray spectroscopy (EDX, X-Max 20, Oxford Instruments, Abingdon, UK) (Figure 1A and B). The XRD spectrum showed a broad halo at 2θ between 20 and 30°, which indicated an amorphous phase [16].

The experimental 1.1 wt% NaF toothpaste in the gel formula was provided by Dentalife (Melbourne, Australia). The actual composition was not provided by the manufacturer. This stock toothpaste was added with varying concentrations of Sr/F-BAGs to produce an experimental formulation. These experimental formulations were prepared fresh prior to each daily treatment. The commercial 1.1.% NaF toothpaste, PreviDent (PV, PreviDent 5000 Plus, Colgate, New York, NY, USA), served as a comparison group (Table 1). All testing materials were stored at 4°C. Five experimental 1.1 wt% NaF toothpastes for the current study are provided below.

**Table 1:** The composition of commercial 1.1% NaF toothpaste.

Materials	Composition	Supplier
Colgate PreviDent 5000 Plus toothpaste (PV)	Purified water, sorbitol, hydrated silica, PEG-12, tetrapotassium pyrophosphate, sodium lauryl sulfate, mint flavour, xanthan gum, sodium benzoate, sodium saccharin, titanium dioxide,	Colgate-Palmolive, New York City, NY, USA

- Group 1: Prevident (PV)
- Group 2: 1.1% NaF experimental toothpaste added with 0 wt% Sr/F-BAG (0% Sr/F-BAG)
- Group 3: 1.1% NaF experimental toothpaste added with 1 wt% Sr/F-BAG (1% Sr/F-BAG)
- Group 4: 1.1% NaF experimental toothpaste added with 2 wt% Sr/F-BAG (2% Sr/F-BAG)
- Group 5: 1.1% NaF experimental toothpaste added with 4% Sr/F-BAG (4% Sr/F-BAG)

These concentrations of bioactive glass (up to 4 wt% Sr/F-BAG) were chosen based on pilot studies that showed no apparent irritation or excessive abrasiveness. The maximum concentration of 4 wt% may not cause excessive abrasiveness in the toothpaste. These formulations were intended for patients at high risk of caries and sensitive oral mucosa, such as those who have undergone head and neck radiation treatment or medications that cause dry and sensitive oral mucosa or oral mucositis [18].

### Measurement of pH, fluoride, and other elemental release (Na, Ca, P, Sr)

Fluoride ion concentrations (ppm) and pH measurements were conducted using a fluoride ion-specific electrode and pH meter (Orien Versastor Pro, Thermos Fisher Scientific, Waltham, MA, USA). The fluoride electrode was calibrated using standard solutions of 0.1,1,10, and 100 ppm, while pH calibration was performed at pH 4, 7, and 10. For analysis, toothpaste samples (100 mg) were diluted in 10 mL of deionised water (*n* = 3). Fluoride concentrations and pH measurements were performed in triplicate (*n* = 3). The diluted solutions were mixed with TISAB II in a 1:1 volume ratio for fluoride determination.

In addition, the elemental analysis of the diluted solutions (*n* = 3) was performed using inductively coupled plasma optical emission spectroscopy (ICP-OES, Optima 8300, PerkinElmer, Waltham, MA, USA) to determine the released concentrations (mg/L) of sodium (Na), calcium (Ca), phosphorus (P), and strontium (Sr). Data analysis was conducted using Syngistix TM for ICP software version 2.0 (PerkinElmer, Waltham, MA, USA). The detection limits were established at 0.5–10 mg/L for Na (589.592 nm), 0.1–10 mg/L for both Ca (317.933 nm) and P (213.617 nm), and 0.1–50 mg/L for Sr (460.733 nm).

### Assessment of dentin remineralisation

The protocol for this investigation was approved by the Human Research Ethics Committee of Thammasat University (Science), Thailand (COE No.031/2567). Extracted human maxillary or mandibular third molars were obtained from the Postgraduate Dental Clinic, Faculty of Dentistry, Thammasat University, Pathum Thani, Thailand. The consent form for collecting the teeth was waived by the committee because the patient’s identification is not required. Only sound teeth without visible caries, crack lines, or hypoplasia were included in the study.

**Figure 2:**
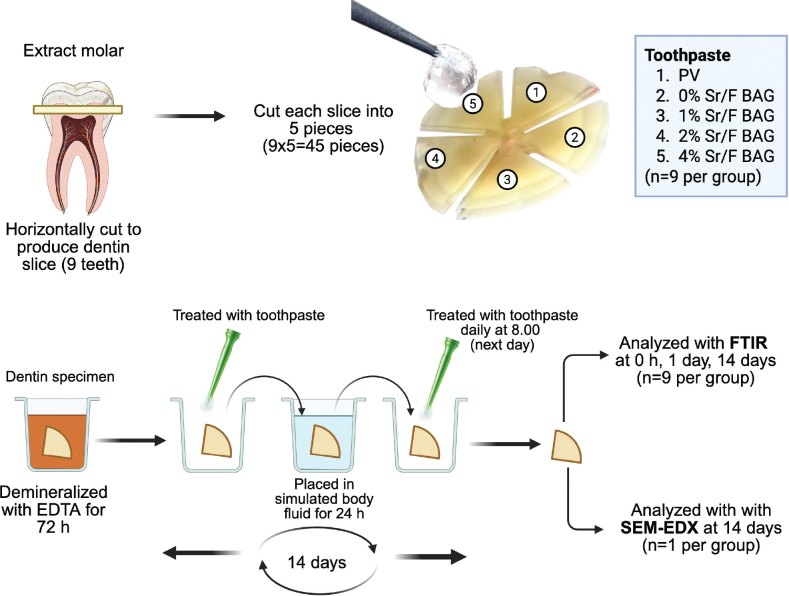
Experimental setup of the remineralisation test of the current study. Created in BioRender. Panpisut, P. (2025) https://BioRender.com/l11a866.

The experimental setup of the remineralisation test is provided in Figure 2. Nine extracted third molars were cleaned and kept in 1% thymol solution (Faculty of Dentistry, Mahidol University, Bangkok, Thailand) at room temperature for a maximum of 3 months before the experiment. Dentin discs of 2 mm thickness were prepared by horizontally sectioning the coronal portion of each tooth using a cutting machine. The dentin discs were subsequently divided into five pieces, each piece from each dentin slice was then randomised for each material (total of 45 pieces, *n* = 9 per group). Then, the specimens were subjected to the 17% ethylenediamine-tetraacetic solution (EDTA, Faculty of Dentistry, Mahidol University, Bangkok, Thailand) and incubated at 37°C for 72 h. This demineralisation protocol was adapted from the previous studies [19, 20], and was reported in a pilot study that showed no mineral detection.

Demineralised dentin specimens were treated with either the experimental 1.1% NaF toothpaste or commercial toothpaste for a 2-min application using an agitating technique with a micro-brush. Excess toothpaste was removed after application, and specimens were immersed in 5 mL of simulated body fluid (SBF), prepared according to BS ISO 23317:2014 Implants for surgery: *In vitro* evaluation for apatite-forming ability of implant materials [21]. The treatment with toothpaste was performed daily for 14 days. The use of SBF was designed to mimic the exposure of applying toothpaste to demineralised dentin and may be exposed to dentinal fluid. The storage solution was replaced daily, and specimens were incubated at 37°C.

Remineralising action was assessed with the Attenuated Total Reflectance Fourier Transform Infrared Spectroscopy (ATR-FTIR, Nicolet iS5, Thermo Fisher Scientific, Massachusetts, USA). The FTIR spectra in the 700–4,000 cm^–1^ region were observed at a resolution of 8 cm^–1^ and 16 repetitions. Data were collected initially, after demineralisation, and after remineralisation for 24 h, 7 days, and 14 days. The peak height at 1,024 cm^–1^ (Abs1024, phosphate group) and 1,636 cm^–1^ (Abs1636, Amide I) absorbance of FTIR spectra were used in this study to represent the phosphate of the apatite [22] and collagen of the organic substance [23–25] in the dentin, respectively. The percentage change in mineral precipitation was determined using the mineral-to-collagen ratio (Abs_1024_/Abs_1636_).

After 14 days, the specimen (*n* = 1) from each group was selected for qualitative investigation of the morphology of the dentin surface. The surface of the dentin specimen was sputter-coated with gold at a current of 23 mA for 45 s using the sputter-coated machine (Q150R; Quorum Technologies, East Sussex, UK). Then, they were examined under a scanning electron microscope (SEM, JSM 7800F, JOEL, Tokyo, Japan) operating at an accelerated voltage of 5 kV. Elemental composition analysis mapping of the surface was performed using Energy Dispersive X-ray Spectroscopy (EDX) to determine the composition of Ca, P, and F of the surface.

### The assessment of in vitro cytotoxicity

L929 mouse fibrosarcoma was donated by Dr. Jasadee Kaewsrichan from the Drug Delivery System Excellence Center, Faculty of Pharmaceutical Sciences, Prince of Songkla University, Thailand. The cells were cultured in 96-well plates at a density of 8 × 10^3^ cells per well, and maintained at 37°C in a humidified incubator with a 5% CO_2_ atmosphere for 24 h. The culture medium consisted of Dulbecco’s Modified Eagle Medium (DMEM; Gibco, Thermo Fisher Scientific, Grand Island, NY, USA) supplemented with 10% foetal bovine serum, 1% penicillin/streptomycin, and 1% L-glutamine (all from Gibco, Thermo Fisher Scientific, Waltham, MA, USA). For cytotoxicity assessment, the experimental toothpaste (*n* = 3) was prepared as a 20% (v/v) dilution in DMEM and filter sterilised. The cells were exposed to 100 µL of this preparation for 2 min, which was the common brushing time [26–29]. The plain culture medium was used as a negative control. Following exposure, the medium was replaced with 0.5 mg/mL MTT solution (Invitrogen, Thermo Fisher Scientific, MA, USA) and incubated for 30 min. The reaction was subsequently terminated by adding 100 µL of dimethyl sulfoxide (Sigma-Aldrich, Saint Louis, MO, USA). Absorbance values were measured at 570 nm and 650 nm wavelengths (OD, optical density) using a microplate reader (Varioskan LUX Multimode, Thermo Fisher Scientific, Waltham, MA, USA) to quantify the final product’s colour. Cell viability was expressed as a percentage relative to the negative control using the following equation. All assays were performed in triplicate.

$$\text{Relative}\;\text{cell}\;\text{viability}=\frac{\text{OD}\;\text{of}\;\text{the}\;\text{test}\;\text{group}}{\text{OD}\;\text{of}\;\text{the}\;\text{control}}\times 100$$ Equation 1

### Statistical analysis

Sample size was estimated using G*Power 3.1 Software (University of Dusseldorf, Germany) based on pilot test results and previously published data [13]. The analysis indicated that sample sizes of *n* = 8 for elemental release and pH, *n* = 9 for remineralising actions, and *n* = 3 for cytotoxicity testing would provide statistical power exceeding 0.95 at α = 0.05 for between-group mean comparisons.

Data were presented as mean and standard deviation (SD) for normally distributed results (pH, fluoride release, elemental release, cell viability) and as median with range (minimum-maximum) for non-normally distributed results (mineral-to-collagen ratio). The analysis was performed using Prism version 10.5.0 for Mac OS (GraphPad Software, San Diego, CA, USA). The Shapiro-Wilk test was used to assess the normality of data distribution. For analysis of mineral-to-collagen ratio, the Friedman test, followed by Dunn’s multiple comparisons, was employed to evaluate changes in the ratio within each group. Between-group comparisons were conducted using the Kruskal-Wallis test followed by Dunn’s procedure. Final changes in mineral-to-collagen ratio and cytotoxicity data were analysed using one-way ANOVA (Analysis of Variance) with post-hoc Tukey HSD (Honestly Significant Difference) test. Statistical significance was established at *p* = 0.05.

## Results

### Measurement of pH, fluoride, and other elemental release (Na, Ca, P, Sr)

**Figure 3:**
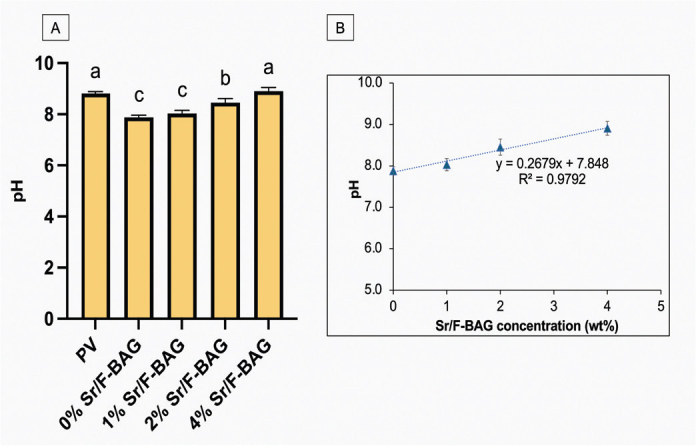
(A) The pH of toothpaste in deionised water. (B) The pH of experimental toothpaste against the concentration of Sr/F-BAG. Error bars are standard deviation (SD) (*n* = 8). Same letters indicate *p* > 0.05.

**Figure 4:**
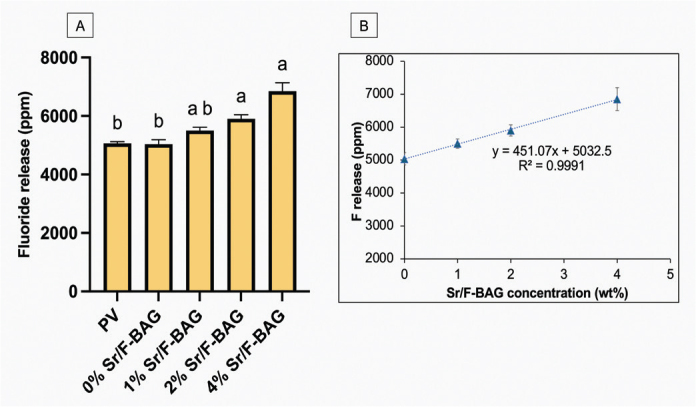
(A) The readily fluoride release of toothpaste in deionised water. (B) The fluoride release of experimental toothpaste against the concentration of Sr/F-BAG. Error bars are standard deviation (SD) (*n* = 8). Same letters indicate *p* > 0.05.

The highest pH was detected in PV (8.8 ± 0.1), which was significantly higher than that of 0% Sr/F-BAG (7.8 ± 0.1, *p* < 0.01), 1% Sr/F-BAG (8.0 ± 0.2, *p* < 0.01), and 2% Sr/F-BAG (8.5 ± 0.2, *p* < 0.01) (Figure 3A). The pH of 4% Sr/F-BAG (8.9 ± 0.2) was comparable to that of PV (8.8 ± 0.1) (*p* = 0.677), which was significantly higher than that of 0% Sr/F-BAG (*p* < 0.01), 1% Sr/F-BAG (*p* < 0.01), and 2% Sr/F-BAG (*p* < 0.01). The pH of experimental toothpaste was linearly increased upon the addition of Sr/F-BAG (*R*^2^ = 0.9792) (Figure 3B). The highest level of fluoride release was detected with 4% Sr/F-BAG (6,858 ± 364 ppm), which was significantly PV (5,063 ± 74 ppm) (*p* < 0.01) and 0% Sr/F-BAG (5,038 ± 185 ppm) (*p* < 0.01) (Figure 4A). The increase of Sr/F-BAG concentration also linearly increased the level of fluoride release from the experimental toothpaste (*R*^2^ = 0.9991) (Figure 4B).

The experimental toothpaste (4% Sr/F-BAG) exhibited the highest level of Ca, Na, P, and Sr release compared to other materials (Table 2). Sr level from PV and 0% Sr/F-BAG was lower than the detection limit of the instrument.

**Table 2:** The concentration of elemental release into deionised water (mean and SD, *n* = 3).

Elements/Group	PV	0% Sr/F-BAG	1% Sr/F-BAG	2% Sr/F-BAG	4% Sr/F-BAG
Ca (ppm)	0.13 (0.04)	0.05 (0.06)	0.99 (0.94)	1.49 (0.2)	2.02 (0.39)
Na (ppm)	94.29 (3.61)	100.98 (5.38)	100.76 (4.1)	104.66 (2.42)	114.72 (9.62)
P (ppm)	51.28 (0.91)	0.43 (0.02)	0.59 (0.05)	0.58 (0.02)	0.62 (0.01)
Sr (ppm)	NA	NA	1.07 (0.32)	1.4 (0.29)	1.9 (0.32)

NA represent the value was under the detection limit of the instrument.

SD: standard deviation; Sr/F-BAG: Sr/F-bioactive glass nanoparticles; PV: Colgate PreviDent 5000 Plus.

### Assessment of dentin remineralisation

**Figure 5:**
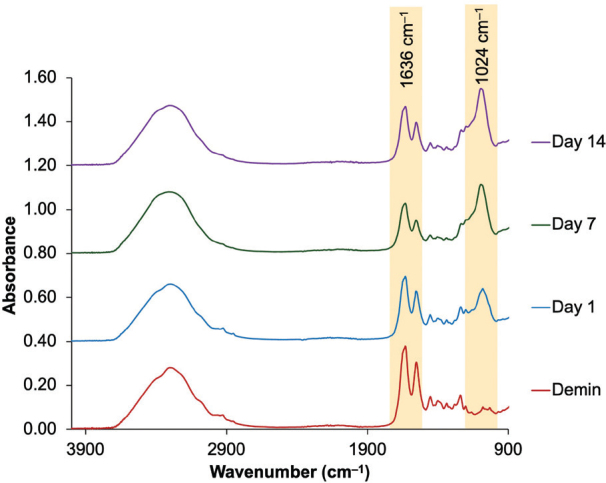
The example of an FTIR result from the representative specimen in the PV group. The height at ~1,030–1,024 cm–1 and ~1,636 cm–1 represents asymmetric stretching vibration of the phosphate group of hydroxyapatites and stretching vibration of the peptide carbonyl group (–C = O) of amide I from collagen.

**Figure 6:**
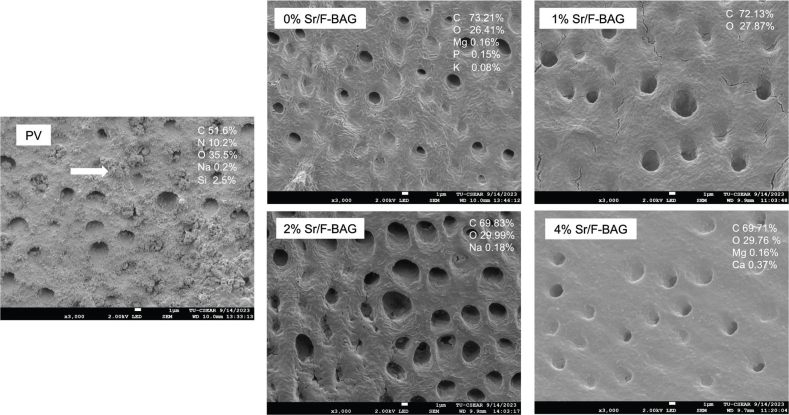
SEM images and EDX results of dentin specimens after 2 weeks. The precipitates containing silica occluding dental tubules (arrow) were detected with PV.

The increase of the mineral-to-collagen ratio (median, mix to max) of PV was significantly increased from 0.041 (0.020–0.061) at 0h to 1.652 (1.058 to 2.390, *p* = 0.0049) and 1.835 (1.136 to 2.918, *p* < 0.01) at 1 week and 2 weeks, respectively (Figure 5, Table 3). However, the mineral-to-collagen ratio of all experimental materials demonstrated no significant increase in the ratio from 0h to 2 weeks (*p* > 0.05). At the final time point, the mineral-to-collagen ratio of 0% Sr/F-BAG (0.041, 0,013 to 0.057), 1% Sr/F-BAG (0.039, 0.009 to 0.054), 2% Sr/F-BAG (0.036, 0.028 to 0.062), and 4% Sr/F-BAG (0.037, 0.029 to 0.058) were comparable (*p* > 0.05). The ratio at 2 weeks of PV was significantly higher than 0% Sr/F-BAG (*p* = 0.002), 1% Sr/F-BAG (*p* = 0.001), 2% Sr/F-BAG (*p* = 0.003), and 4% Sr/F-BAG (*p* = 0.006). The dentin surface of specimens treated with PV showed substantial precipitation of mineral on the surface (Figure 6). However, the surface of dentin treated with experimental 1.1% NaF toothpaste showed minimal precipitation compared with PV.

**Table 3:** The Mineral-to-collagen ratios (median) obtained from specimens in each group for up to 2 weeks (*n* = 9).

Group/time	Demin	1 Day	7 days	14 days
**PV**	0.041	0.041	1.652	1.835
	(a)(B)	(a)(B)	(a)(A)	(a)(A)
**0%** Sr/F-BAG	0.046	0.046	0.041	0.037
	(a)(A)	(a,b)(A)	(b)(A)	(b)(A)
**1%** Sr/F-BAG	0.036	0.036	0.039	0.036
	(a)(A)	(b)(A)	(b)(A)	(b)(A)
**2%** Sr/F-BAG	0.046	0.046	0.036	0.036
	(a)(A)	(b)(A)	(b)(A)	(b)(A)
**4%** Sr/F-BAG	0.037	0.037	0.037	0.042
	(a)(A)	(b)(A)	(b)(A)	(b)(A)

Lower-case letters indicate *p* > 0.05 within the same column, while upper-case letters indicate *p* > 0.05 within the same row.

Sr/F-BAG: Sr/F-bioactive glass nanoparticles; PV: Colgate PreviDent 5000 Plus.

**Figure 7:**
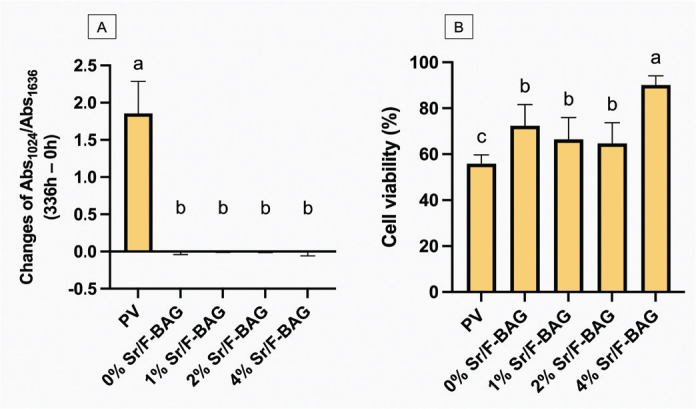
A) The total changes of the mineral-to-collagen ratio of specimens treated with different toothpastes at 14 days, compared with after demineralisation. Error bars are standard deviation (SD) (*n* = 9), same letters indicate *p* > 0.05. (B) The cell viability after being treated with different toothpastes. Error bars are SD (*n* = 3), same letters indicate *p* > 0.05.

When comparing the total change in the mineral-to-collagen ratio at 2 weeks to that at the initial time (Figure 7), the ratio of PV (1.812, 1.116 to 2.877) was significantly higher than that of the other materials (*p* < 0.05). The total change of 4% Sr/F-BAG (0.006, -0.139 to 0.048) was comparable to that of 0% Sr/F-BAG (-0.010, -0.090 to 0.045), 1% Sr/F-BAG (0.005, -0.022 to 0.008), and 2% Sr/F-BAG (-0.003, -0.021 to 0.041) (*p* > 0.05).

### The assessment of in vitro cytotoxicity

The highest cell viability was detected with 4% Sr/F-BAG (90 ± 2%), which was significantly higher than PV (56 ± 16%) and other experimental materials (*p* < 0.05) (Figure 7B). The value of 0% Sr/F-BAG (72 ± 4%) was similar to that of 1% Sr/F-BAG (67 ± 4%) (*p* = 0.204), and 2% Sr/F-BAG (65 ± 4%) (*p* = 0.071).

## Discussion

This study examined the effect of the concentration of Sr/F-BAG incorporated into 1.1% NaF toothpaste. The addition of Sr/F-BAG and its concentration affected fluoride release, elemental release, pH, and *in vitro* cytotoxic effects, but not the remineralising effects. Hence, the null hypothesis of the current study was partially rejected.

The increase in the concentration of Sr/F-BAG enhanced the release of multiple elements. This observation is consistent with previous research indicating that F-bioactive glass incorporated into toothpaste interacts with solutions, leading to its degradation and gradual release of ions such as calcium, phosphate, and fluoride [30]. Furthermore, the degradation of the glass is typically associated with an increase in pH, which could explain the observed rise in pH for the experimental materials [31]. Linear regression analysis from the current study suggests that with a 1 wt% increase in Sr/F-BAG, the pH and fluoride release levels for the toothpaste would increase by approximately 0.3 and 457 ppm, respectively. These findings could potentially assist in optimising formulations for oral health products.

The study found that the experimental 1.1% NaF toothpaste exhibited minimal changes in dentin remineralisation in comparison to the commercial toothpaste. It is important to note that the manufacturer did not provide the actual composition of the base-toothpaste used to prepare the experimental toothpastes. This lack of information is a limitation for further interpretation of the results. Therefore, the findings should be interpreted with caution.

It is challenging to identify the actual mechanism for the lack of remineralising effects of experimental 1.1 wt% NaF toothpaste in the current study. One of the possible causes may be because of the use of carboxymethyl cellulose (CMC). It was expected that CMC could be the main gelling agent used for experimental toothpaste in the gel form. It was reported that CMC contains carboxyl groups that can interact with metal ions through electrostatic interactions and complexation [32, 33]. Hence, CMC with multiple active sites may promote adsorption with Ca or Sr ions, and subsequently reduce the diffusion of ions into the underlying demineralised dentin [34, 35]. This could potentially lead to the inferior remineralising effects of the experimental toothpaste. However, future work will require a negative control (formulation with no CMC) to confirm this hypothesis.

The commercial toothpaste contained silica particles that may be incorporated into demineralised dentin. This may promote tubule occlusion effects and provide irregularities of the surface that may promote adsorption of minerals to prevent further demineralisation. The crystals in the silica/collagen network may help promote nucleation for apatite in demineralised dentin [36], which may result in a higher increase in mineral-to-collagen ratio.

It was expected that the increase in peak representing hydroxyapatite observed from the FTIR result of PV should result in an increase in Ca and P composition detected by SEM-EDX. However, the amount of Ca and P was not clearly detected. This finding was consistent with the previous study, which demonstrated that the PV promoted substantial precipitation of silica-based compounds occluding dentinal tubules in irradiated, demineralized dentin [13]. It was hypothesized that silica compounds may be more dominant than Ca and P from apatite, thereby limiting the detection of Ca and P through EDX analysis. The previous study demonstrated that the application of bioactive glass nanoparticles (particle size of 20 nm and surface area of ~ 63 ± 0.2 m^2^/g) on demineralised dentin resulted in intensified diffraction peaks at 26° and 32°, suggesting the formation of hydroxyapatite (HA) [37]. The absence of confirmation from X-ray diffraction (XRD) analysis is a limitation of the current study. Therefore, future studies should incorporate an additional analytical method, such as XRD, to comprehensively analyse the presence of hydroxyapatite in the specimen.

The current study utilised SBF as the storage solution to preliminarily assess the remineralising effects of the toothpaste on dentin. It was anticipated that the toothpaste would interact with the dentinal fluid within the tubules [38]. However, a standardised formulation for simulated dentinal fluid has not yet been established. Consequently, we prepared the fluid following the protocol for SBF preparation, which may have a similar composition to other bodily fluids [21]. Therefore, the results should be interpreted with caution. Future work should employ the pH cycling method to assess the remineralising effects of toothpaste upon biofilm challenge, which is more clinically relevant.

The lowest cell viability was observed with PV, potentially because of the surfactant, such as sodium lauryl sulfate (SLS), present in PV. The previous study indicated that SLS induced a cytotoxic effect in mouse fibroblasts by inhibiting cell growth and enhancing apoptosis in a dose-dependent manner [39], and also increased the risk of mucosal irritation [40]. Increasing BAG from 1 to 4 wt% could enhance cytocompatibility of the experimental toothpaste (cell viability > 70% [41]). It was reported that the increase in Sr in the composition of bioactive glass resulted in improved cell viability (> 85%) [42]. It was reported that Sr-doped BAG, such as Sr/F-BAG, may promote Sr^2+^ release at concentrations beneficial for cells, supporting cell proliferation and osteogenic differentiation while avoiding cytotoxic levels [43, 44]. The actual mechanisms of the beneficial effects of Sr-doped BAG have not yet been concluded. The addition of Sr^2+^ reduced the degradation rate of bioactive glass compared with the pure bioactive glass, which may facilitate the sufficient time required for extracellular matrix formation [45]. Furthermore, the internalised Sr^2+^ interacts with the intercellular signalling molecules to stimulate biological functions [46].

The study indicates that producing 1.1% NaF toothpaste with bioactive glass can increase fluoride release and elevate pH levels, which may aid in caries prevention. Local production of this toothpaste could help overcome the challenges related to importing biomedical devices for dental care in some countries [6]. The optimisation of the formulation is needed to consider the cost-benefit for the use of reactive fillers. Although the remineralising effects of the experimental 1.1% NaF toothpaste were not detected in this *in vitro* study, it is important to acknowledge its limitations and consider future testing. This preliminary test aimed to determine a suitable concentration of bioactive glass that could be incorporated into the toothpaste. A high concentration may promote fluoride release but may additionally result in increased abrasiveness and higher unit costs. Using a lower concentration (1–2 wt%) may be recommended to reduce abrasiveness, particularly for patients with highly sensitive oral mucosa. However, determining the ideal concentration based solely on remineralising studies may not be sufficient. Future research should include caries models using a biofilm model, which is more relevant to better understand the effects of high pH, fluoride ions, and bioactive glass on biofilm modulation. The abrasiveness of the toothpaste to enamel or dentin should also be determined in the future study.

## Conclusion

The commercial 1.1% NaF toothpaste demonstrated the highest remineralising effects. Increasing the concentration of Sr/F-BAG enhanced pH levels, fluoride, and other elemental release, in addition to improving the *in vitro* cytocompatibility of the experimental 1.1% NaF toothpaste. However, the concentration of bioactive glass nanoparticles had a minimal effect on the remineralising potential of the toothpaste on demineralised dentin.

## Data Availability

The data that support the findings of this study are available from the corresponding author upon reasonable request.
